# Chemokine-mediated distribution of dendritic cell subsets in renal cell carcinoma

**DOI:** 10.1186/1471-2407-10-578

**Published:** 2010-10-22

**Authors:** Peter Middel, Sven Brauneck, Werner Meyer, Heinz-Joachim Radzun

**Affiliations:** 1 Institut für Pathologie Nordhessen, Germaniastrasse 7-9, 34119 Kassel, Germany; 2 Klinische Forschergruppe 179, Universitätsmedizin Göttingen, Robert-Koch-Str. 40, 37075 Göttingen, Germany; 3 Zentrum für Pathologie, Universitätsmedizin Göttingen, Robert-Koch-Str. 40, 37075 Göttingen, Germany

## Abstract

**Background:**

Renal cell carcinoma (RCC) represents one of the most immunoresponsive cancers. Antigen-specific vaccination with dendritic cells (DCs) in patients with metastatic RCC has been shown to induce cytotoxic T-cell responses associated with objective clinical responses. Thus, clinical trials utilizing DCs for immunotherapy of advanced RCCs appear to be promising; however, detailed analyses concerning the distribution and function of DC subsets in RCCs are lacking.

**Methods:**

We characterized the distribution of the different immature and mature myeloid DC subsets in RCC tumour tissue and the corresponding normal kidney tissues. In further analyses, the expression of various chemokines and chemokine receptors controlling the migration of DC subsets was investigated.

**Results:**

The highest numbers of immature CD1a+ DCs were found within RCC tumour tissue. In contrast, the accumulation of mature CD83+/DC-LAMP+ DCs were restricted to the invasive margin of the RCCs. The mature DCs formed clusters with proliferating T-cells. Furthermore, a close association was observed between MIP-3α-producing tumour cells and immature CCR6+ DC recruitment to the tumour bed. Conversely, MIP-3β and SLC expression was only detected at the tumour border, where CCR7-expressing T-cells and mature DCs formed clusters.

**Conclusion:**

Increased numbers of immature DCs were observed within the tumour tissue of RCCs, whereas mature DCs were found in increased numbers at the tumour margin. Our results strongly implicate that the distribution of DC subsets is controlled by local lymphoid chemokine expression. Thus, increased expression of MIP-3α favours recruitment of immature DCs to the tumour bed, whereas *de novo *local expression of SLC and MIP-3β induces accumulation of mature DCs at the tumour margin forming clusters with proliferating T-cells reflecting a local anti-tumour immune response.

## Background

Dendritic cells (DCs) are the most potent antigen-presenting cells (APCs), and play a central role in the processing and presentation of antigens to T cells during an immune response [1]. DC progenitors in the bone marrow give rise to circulating precursors that home to the tissue where they reside as immature cells with high phagocytic capacity. Upon tissue damage or exposure to antigens, DCs capture antigens and subsequently migrate to the lymphoid organs, where they select the rare antigen-specific T cells and initiate a cellular immune response [1,2]. It has been shown that during migration and within secondary or tertiary lymphoid organs, DCs undergo functional maturation from antigen collection and processing to very potent APCs [1,3,4]. Immature DCs capture antigens, but weakly stimulate T lymphocytes. In the presence of particular signals, such as lipopolysaccharide (LPS) or various cytokines, immature DCs mature into potent T stimulatory cells, a process that is associated with up-regulation of co-stimulatory molecules (CD80, CD86, CD40, CD83, and DC-LAMP), as well as changes in chemokine receptors expressed on their surface [1-6]. Immature CD1a+ DCs are CC-chemokine-receptor (CCR) 6-positive and respond to MIP-3α [7]. In contrast, mature DCs are attracted by the chemokine, MIP-3ß, or secondary lymphoid chemokines (SLCs) following *de novo *expression of CCR7 [6,8,9]. A critical characteristic of fully mature DCs is the production of pro-inflammatory cytokines, particularly IL-12, which plays a critical role in the induction of efficient T-helper cell 1 immunity [10], as observed for an efficient anti-tumour T cell response [1,6].

The involvement of DCs in tumour immunity has clinical importance. The infiltration of DCs into some primary tumour types has been found to be associated with significantly improved patient survival and a reduced incidence of recurrent disease [11,12]. It is known that tumours avoid surveillance by the immune system through various mechanisms, including the inhibition of the recruitment of DCs at the tumour site, as well as impairment of function of DCs by local production of immunosuppressive cytokines [13]. However, the precise knowledge of the tumour environment, which varies between different tumour types, might be important for the design of optimal immunotherapeutic strategies against cancer [14-16].

Promising results have been previously reported using DC-based vaccination against immunogenic tumours, such as melanoma or renal cell carcinoma (RCC) [17,18]. A subset of patients with metastatic RCC develops significant immune and clinical responses after immunotherapy with DC vaccination [1]. In this context mature DCs are thought to play a key role, since they are known to represent the most effective antigen presenting cells for induction of a potent T cell response. In order to give an answer to the question why some patients respond to DC-vaccine based therapies and others not, a detailed knowledge about the cellular T cell immune response in RCC with special regard to antigen-presenting DCs is required. However, detailed studies concerning the type and distribution of DC subsets in RCC are still lacking.

More recently, novel markers have emerged allowing the identification of a broader spectrum of DC subpopulations with respect to their function on formalin-fixed and paraffin-embedded tissue. Thus, we characterized the phenotype, distribution, and maturation of the different DC subsets in RCCs. In addition, we analyzed the local expression of chemokines that are known to play a key role for the recruitment of DC subsets.

## Methods

### Tissue samples

Tumours and corresponding tumour-free tissue of nephrectomy specimens from 24 patients with RCCs were included in this study. All tumours were freshly obtained from the Urologic Department's operating room. The patient ages ranged from 36-75 years (median age, 60 years). None of the patients investigated in this study were treated before surgery. The formalin-fixed and paraffin-embedded specimens were cut into 5-μm-thick sections and placed on poly-L-lysine-treated glass slides. Representative sections were stained with routine hematoxylin and eosin (H&E) and evaluated. The histologic types of cancer were as follows: clear cell (n = 17); papillary (n = 4); chromophobe (n = 1); and sarcomatoid renal cell carcinoma (n = 2). This study was approved by the Ethics Committee of Georg-August-University Göttingen, and the Helsinki Declaration regarding the use of human tissue was followed. Informed consent was obtained from the patients for the use of their tissue samples.

### Immunohistochemistry

The antibodies used in the study and the optimal working dilutions are listed in Table 1. The sections were immunostained applying the biotin-streptavidin-peroxidase method (Multi-Link, DCS, Hamburg, Germany). Immunostaining for chemokines (MIP-3α, MIP-3β, and SLC) required application of the tyramide signal amplification system method (NEN, Boston, MA, USA). In control reactions, isotype- and species-specific matched control antibodies were applied.

**Table 1:** Antibodies used for immunohistochemistry and immunofluorescence (Ms - mouse, Rb - rabbit antibody).

Antibody	Dilution	Species	Vendor
CD1a	1:50	Ms	LabVision, Fremont, CA, USA

CD3	1:50	Ms	Novocastra, Newcastle, UK

CD4	1:50	Ms	DAKO, Hamburg, Germany

CD11c	1:50	Ms	DAKO

CD40	1:50	Ms	Acris, Hiddenhausen, Germany

CD68	1:50	Ms	DAKO

CD79a	1:50	Ms	DAKO

CD83	1:20	Ms	Novocastra

DC-LAMP/CD208	1:50	Ms	Acris

Langerin	1:50	Ms	Acris

RelB	1:200	Rb	SantaCruz

CCR6	1:20	Ms	R&D Systems

CCR7	1:20	Ms	R&D Systems

MIP-3α	1:20	Goat	R&D Systems

MIP-3β	1:20	Goat	R&D Systems

SLC	1:50	goat	R&D Systems

Vimentin	1:100	Ms	DAKO

Fascin	1:100	Ms	DAKO

D2-40	No	Ms	DCS-Diagnostics, Hamburg, Germany

Ki-67	1:50	Ms	DAKO

For double immunofluorescence staining, the slides were stained for 1 hour with unconjugated primary antibody, followed by incubation with indocarbocyanine 2 (Cy2)-conjugated goat anti-mouse or goat anti-rabbit F(ab)-fragments (both from Dianova, Hamburg, Germany) at a saturating concentration for 60 minutes. For the first step in immunofluorescence staining for MIP-3α, MIP-3β, and SLC, we adopted the TSA-Kit (NEN) using FITC-conjugated tyramide for fluorescence amplification. Sections were washed and incubated with the second antibody for 60 minutes at room temperature, followed by incubation with indocarbocyanine 3 (Cy3)-conjugated goat anti-mouse or goat anti-rabbit F(ab)-fragments (both from Dianova) for 60 min at room temperature.

Confocal fluorescence images were obtained on a Leica TCS (Leica Microsystems, Heidelberg, Germany) confocal system mounted on an Olympus BX50 WI microscope (Tokyo, Japan). Possible cross-talk between FITC or Cy2 and Cy3, which could give rise to false-positive co-localization of different signals, was avoided by careful selection of the imaging conditions.

### RT-PCR analysis

Sequences of primers used in the study are listed in Table 2. Primers and probes (Operon-Qiagen, Hilden, Germany) were designed using the Primer-3 online primer design program http://www-genome.wi.mit.edu. Optimal conditions for all primers were established by amplifying cDNA samples from human tonsil or lymph node.

**Table 2:** PCR primers used for RT-PCR analysis of chemokines

Gene	Primer sequence	Product size
MIP-3α	5'- CTGTACCAAGAGTTTGCTCC -3'	193bp
	5'- GCACAATATATTTCACCCAAG -3'	

MIP-3β	5'-CCAGCCTCACATCACTCACACCTTGC-3'	324 bp
	5'-TGTGGTGAACACTACAGCAGGCACCC-3'	

SLC	5'-AACCAAGCTTAGGCTGCTCCATCCCA-3'	249 bp
	5'-TATGGCCCTTTAGGGGTCTGTGACCG-3'	

β-Actin	5'- CTACAATGAGCTGCGTGTGGC -3'	270 bp
	5'- CAGGTCCAGACGCAGGATGGC -3'	

Total cellular mRNA was extracted with the RNeasy Mini Kit (Operon-Qiagen). RNA integrity and quantity was assessed using the Agilent Bio-analyzer 2100 (Agilent Technologies, Waldbronn, Germany). Reverse transcription with random hexamer primers was performed with the Omniscript RT Kit (Qiagen). Quantification of MIP-3α, MIP-3β, SLC, and ß-actin mRNA expression was performed on an iCycler iQ real-time detection system (Bio-Rad, Hercules, CA, USA) using the HotStar *Taq*DNA polymerase kit (Qiagen). Expression of MIP-3α, MIP-3β, and SLC was normalized to ß-actin expression to compensate for different sample capacities. Results derived from the PCR standard curve are given in attomoles per μg of total cellular RNA. cDNA from a human lymph node was used as a positive control template for each primer pair. Negative controls with water instead of cDNA were always included.

### Tumour cell lines

The RCC cell lines (A498, Caki-1, and Caki-2) were purchased from the Deutsche Sammlung von Mikroorganismen und Zellkulturen (DSZM, Braunschweig, Germany). All 3 cell lines were cultivated at 37°C in 5% CO_2 _in RPMI-1640 containing 10% fetal calf serum, 10 mm l-glutamine, 1% penicillin/streptomycin, 2.5% HEPES buffer, and 1% amino acid solution.

### Statistics

The evaluation of immunoreactivity was performed on sections stained for CD1a and CD83. Because of the heterogeneous distribution of DC, the quantification was performed by the hot-spot method. This method allows quantification of cells in hot spots defined in this study as areas containing the highest density of positive cells. Accordingly, five hot spots per section were counted with an eyepiece graticule at 400x magnification. Statistical analyses were applied using GraphPad Prism (version 5.00 for Mac; GraphPad Software, San Diego, CA, USA). Statistical comparisons were performed applying the unpaired t-test or the Mann-Whitney test; p values < 0.05 were considered as statistically significant.

## Results

### Immature DCs are present within the tumour tissue

**Figure 1:**
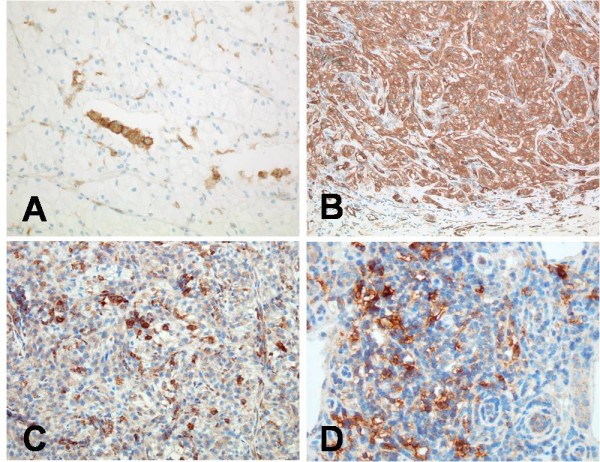
**Immunohistochemistry for different DC markers**. CD11c is co-expressed by numerous macrophages within the tumour tissue (A, 200x). Moderate-to-strong Fascin expression by tumour cells of RCC (B, 100x). Numerous CD1a+ immature DCs can be observed within the tumour stroma (C, 200x). CD83+ mature dendritic cells are observed within T-cell aggregates, which are located adjacent to the infiltration border of the tumour cells (D, 200x).

To evaluate the total number of DCs in tumour tissues of RCCs and the corresponding normal kidney tissues, immunostaining for CD11c and Fascin was performed. These markers have been shown to be widely expressed by subsets of immature and mature myeloid DCs (19, 20). Unfortunately, both markers proved not to be specific for DCs in renal tissues. For instance, immunostaining for CD11c (Figure 1a) revealed co-expression of this antigen by numerous macrophages, which were predominantly present within the tumour tissue and at the tumour margin. Fascin demonstrated a strong cytoplasmatic co-expression by tumour cells in >90% of the cases investigated (Figure 1b). Fascin staining was found to be heterogeneous within the tumour tissue. Thus, in many cases the strongest cytoplasmatic expression for Fascin was observed by tumour cells localized at the infiltration border. Therefore, evaluation of immature and mature DC subsets was performed by immunhistochemistry for CD1a (Figure 1c) and CD83 (Figure 1d), respectively.

**Figure 2:**
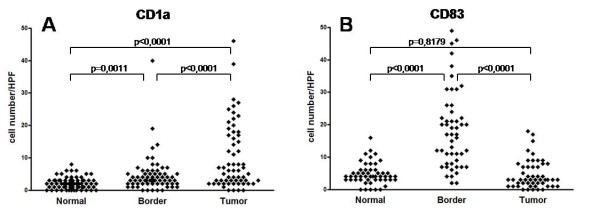
**Quantification of CD1a (A) and CD83 (B) expressing dendritic cells (DCs) in RCC tissue, tumour infiltration border zone and corresponding normal kidney**. (A) Tumour tissue of RCC shows a statistically significant increased number of immature CD1a+ DCs (Mean: 14.44, SD: 19.59) compared to the tumour infiltration border zone (Mean: 4.621, SD: 5.577) and normal kidney tissue (Mean: 2.182, SD: 1.805). (B) In contrast, a significant increased amount of mature CD83+ DCs is observed within the tumour infiltration border zone (Mean: 18.41, SD: 11.94) compared to tumour tissue (Mean: 4.6608, SD: 4.364) and normal kidney (Mean: 4.784, SD: 3.282; Mann-Whitney test with Bonferroni correction; p values < 0.05 were considered as statistically significant).

**Figure 3:**
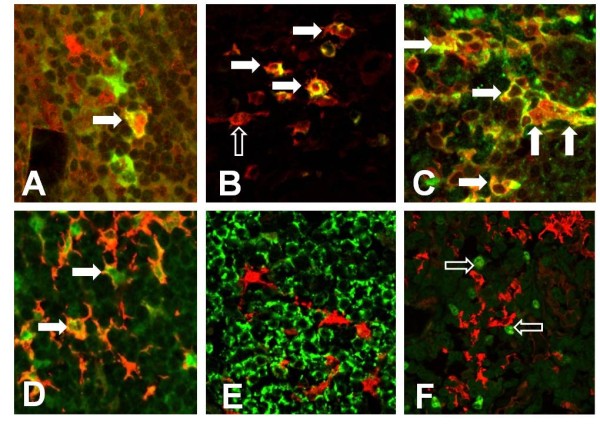
**Characteristics of DC subsets in RCC**. By double immunfluorescence investigations the immature CD1a+ DCs (indocarbocyanine 3 (Cy3) - red fluorescence) in the tumour bed show co-expression for Langerin (indocarbocyanine 2 (Cy2) - green fluorescence; A, 400x). CD83+ DCs (Cy3 - red fluorescence) of the tumour border show co-expression for the maturation-associated antigens DC-LAMP (B), CD40 (C), and nuclear-expressed RelB (D, all 400x, all Cy2 - green fluorescence). CD83+ DCs (Cy3 - red fluorescence) form clusters with CD3+ T-cells (E, 400x, Cy2 - green fluorescence). T cells adjacent to the CD83+ mature DCs (Cy3 - red fluorescence) show an increased proliferation as indicated by nuclear Mib-1 expression (F, 400x, Cy2 - green fluorescence).

Immature DCs were characterized by CD1a expression (Figure 1c), and the LC subset of immature DCs was further characterized by additional expression of Langerin. In all samples, CD1a+ cells with dendritic morphology were found randomly distributed throughout the entire tumour beds. The number of CD1a+ immature DCs per high-power field within the tumour tissue ranged from 1-24 cells, with mean and median numbers of 12 and 7, respectively (Figure 2a). Although a majority of DCs were CD1a+/Langerin+ LCs (Figure 3a), CD1a+/Langerin- cells were also found. Langerin co-expression was observed by 80%-90% of CD1a+ DCs. The immaturity of CD1a+ DCs within the tumour tissue was further demonstrated by the absence of maturity markers, such as DC-LAMP, CD40, or RelB expression, as assessed by serial analyses in confocal microscopy of double fluorescence-stained sections. Normal kidney tissues rarely displayed scattered CD1a+ cells. Three sections were negative, whereas 9 of the 24 sections investigated showed <3 CD1a+ cells within the normal kidney over the entire section. However, in four cases, a weak-to-moderate chronic nephritis was present. In these latter cases, focal aggregates of CD1a+ DCs were observed due to the chronic inflammatory process.

### Mature DCs accumulate in the peri-tumoural area

We next sought to determine if mature DCs were also infiltrating RCC tumour tissues. Mature DCs were characterized by expression of CD83 (Figure 1d). CD83+ mature myeloid DCs could be observed in all tumour cases investigated. However, in all cases, a striking compartmentalization of CD83+ cells was observed. Thus, only rare CD83+ DCs were observed within normal, kidney tissues not affected by inflammation. As a rule, no more than three CD83+ DCs could be observed within the entire normal kidney area of the slides investigated. Even in the tumour tissues, only rare CD83+ DCs were observed. Thus, in 17 of the 24 investigated cases, no CD83+ DCs were identified within the tumour tissue. Most interestingly, numerous mature DCs were located in the peri-tumoural normal kidney tissue in close vicinity to the typically observed pseudocapsule of RCCs (Figure 1d). In the peri-tumoural area, the number of CD83+ mature DCs per high-power field (400x) ranged from 0-28 cells, with mean and median numbers of 6 and 7, respectively (Figure 2b). In addition, double staining for DC-LAMP (Figure 3b), CD40 (Figure 3c), and nuclear RelB (Figure 3d) confirmed the maturity of CD83+ DCs.

### T cells make clusters with mature DCs in the peritumoural area

Because mature DCs can normally be found only in secondary lymphoid organs where they closely interact with Ag-specific T cells, we next analyzed the T cell distribution and activation pattern in RCC samples. In 22 of the 24 evaluated samples, CD3+ T cells infiltrated peri-tumoural areas, where they could be seen either scattered throughout the area in close proximity to tumour cells or clustered. Double-staining for CD3 and CD83 demonstrated T cell clusters around mature DCs in the peri-tumoural areas (Figure 3e). Typically, the DC/T-cell clusters were located adjacent or around peri-tumoural lymphatic vessels. Occasionally, DC/T-cell clusters were found within peri-tumoural lymph vessels. The majority of infiltrating CD3+ T-cells (70%-75%) showed co-expression for CD4. Approximately 5%-10% of T cells within the DC T cell clusters at the tumour border showed signs of proliferation, as shown by nuclear expression of the proliferation marker Ki67 (Mib-1; Figure 3f).

### mRNA expression of lymphoid chemokines in renal cell carcinoma tissue and tumour cell cultures

**Figure 4:**
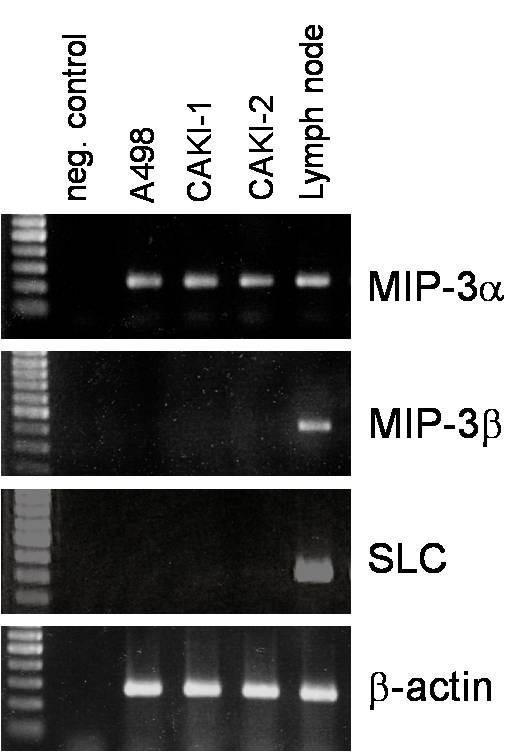
**RT-PCR analysis of MIP-3α, MIP-3β, and SLC mRNA expression in renal carcinoma cell lines**. RT-PCR showed amplification products for MIP-3α in all three RCC cell lines (A498, CAKI-1, and CAKI-2) investigated. In contrast, no amplification products for MIP-3β and SLC were obtained. mRNA derived from lymph node tissue served as positive control. Quality of cDNA was checked by amplification for β-actin.

To explore the differential expression of the lymphoid chemokines MIP-3α, MIP3-β, and SLC in RCC cell lines and RCC tissue RT-PCR analyses were performed. RT-PCR on total mRNA isolated from three established RCC lines (A498, Caki-1, and Caki-2) showed abundant PCR products for MIP-3α by all three cell lines investigated (Figure 4). In contrast, no amplification products could be obtained by RT-PCR for MIP-3β and SLC. mRNA isolated from human lymph nodes served as a positive control, showing gene-specific amplification products for all three genes.

**Figure 5:**
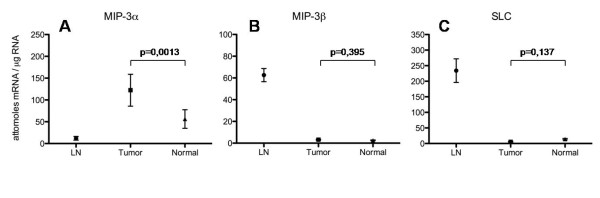
**Real-time RT-PCR analysis of MIP-3α, MIP-3β, and SLC mRNA expression**. Total RNA was analyzed for MIP-3α (A), MIP-3β (B), and SLC (C) mRNA expression by quantitative RT-PCR from tissue samples of RCC and corresponding normal kidney tissues of the same patient (n = 10). mRNA isolated from five normal lymph nodes served as positive controls showing abundant transcripts for MIP-3β and SLC, whereas only weak expression for MIP-3α could be observed. RCC tissue demonstrated a significantly increased expression of MIP-3α mRNA compared to the corresponding normal kidney (p = 0.0013). In contrast MIP-3β (p = 0,395) and SLC (p = 0,137) did not prove to be significantly different between tumour tissue and controls.

Thus, additional studies were performed on the total RNA isolated from the carcinoma tissue and corresponding normal kidney tissue by quantitative real-time RT-PCR (n = 10). For carcinoma-derived mRNA, a statistically significant increased expression of MIP-3α (p < 0.05) mRNA was observed in comparison to mRNA of corresponding normal kidney tissue (Figure 5a). In contrast, no statistical significant difference was detected for MIP-3β (p = 0.395; Figure 5b) and SLC (p = 0.137; Figure 5c) between tumour mRNA and mRNA isolated from corresponding normal kidney tissues.

### Immunohistochemical analysis of MIP-3α, MIP-3β, and SLC in RCC tissue and corresponding normal kidney tissues

**Figure 6:**
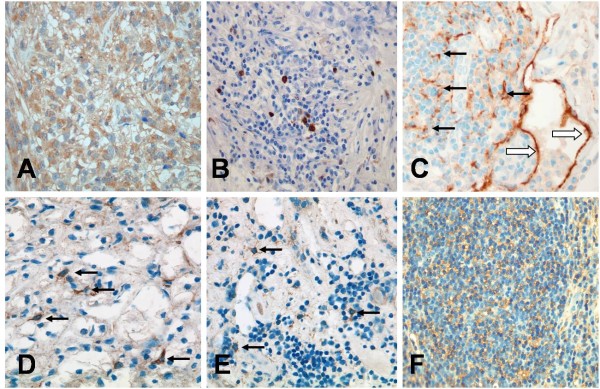
**Immunohistochemistry of chemokine and chemokine receptor expression in RCC**. Tumour cells show moderate cytoplasmatic expression for MIP-3α (A, 400x). Expression of MIP-3β is observed by DCs localized within lymphocyte aggregates at the tumour infiltration border (B, 400x). SLC is expressed by lymphatic vessels (open arrows) and in addition by stromal cells (arrows) at the tumour infiltration border (C, 400x). CCR6 positive cells with dendritic morphology are randomly distributed between the tumour cells (D, 400x). Few CCR6 positive DCs can be observed within the lymphatic aggregates of the tumour border zone (arrows, E, 400x). Numerous CCR7+ cells are found within the lympoid aggregates (F, 400x).

The expression of MIP-3α, MIP3-β, and SLC was then evaluated by immunohistochemistry and double-immunofluorescence on paraffin sections of the cases analyzed by RT-PCR. Immunostaining for MIP-3α was observed within the cytoplasm of tumour cells in all cases investigated (Figure 6a). Furthermore, expression by tumour-associated macrophages was observed, as determined by double-staining with the monocyte/macrophage marker, CD68. A weak-to-moderate immunostaining could also be observed by tubulus cells of normal kidney tissues.

**Figure 7:**
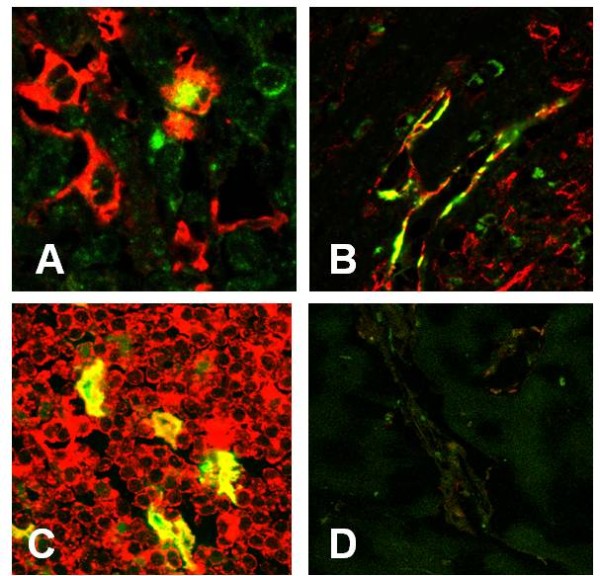
**Two-colour immunofluorescence investigation of chemokine and chemokine receptor expression**. MIP-3β (Cy2 - green fluorescence) is expressed by CD83+ co-expressing DCs (Cy3 - red fluorescence; A, 600x). In contrast, SLC (Cy3 - red fluorescence) is co-expressed by D2-40+ lymphatic endothelial cells (Cy2 - green fluorescence; B, 400x). Within the lymphatic infiltrates of the tumour border zone CCR7 (Cy3 - red fluorescence) is co-expressed by CD83+ mature DCs (Cy2 - green fluorescence; C, 400x). Negative control (D, 400x).

Immunohistochemistry for MIP-3β and SLC demonstrated no staining of epithelial cells, including tumour cells and normal kidney tubules. In addition, no staining was observed by tumour-associated macrophages or other inflammatory cells, such as CD3+ T-cells or CD79a+ B-lymphocytes. In contrast, an increased number of MIP-3β-expressing cells were observed within the T-cell clusters (Figure 6b). The cells expressing MIP-3β proved to be mature myeloid dendritic cells showing co-expression for CD83 (Figure 7a). Similarly, an increased number of SLC-expressing dendritic-shaped cells (Figure 6c) were observed within T cell aggregates often in direct contact to CD11c and CD83-positive DCs. The SLC-expressing cells within these T cell clusters proved to be negative for DC markers (CD1a, CD11c, and CD83), as well as antigens related to T cells (CD3), B-cells (CD79a), or macrophages (CD68, data not shown). Since the SLC-expressing cells demonstrated expression of vimentin (data not shown), they probably represent fibroblastic stromal cells which have already been shown to represent the major source of SLC-expressing cells within the lymph node paracortex. Furthermore, we found an increased number of lymphatic vessels in the peri-tumoural region, whereas the tumours themselves were completely devoid of lymph vessels as determined by immunostaining for the lymphatic endothelial cell marker, D2-40. The lymphatic vessels of the tumour border showed a strong expression for SLC (Figure 6c), as determined by co-expression for D2-40 (Figure 7b). The SLC-expressing lymphatic vessels were typically observed within or adjacent to DC/T-cell clusters. In addition, occasional clusters of T cells and DCs could be detected within these SLC-positive lymphatic vessels.

### Chemokine receptor expression by immature and mature DCs in RCCs

CCR6-positive cells showing dendritic morphology were randomly distributed throughout the tumour bed (Figure 6d) in all cases investigated. CD1a+ DCs within the tumour area and normal kidney tissue demonstrated co-expression for CCR6, whereas no expression for CCR7 could be observed as determined by immunohistochemistry and double-immunofluorescence studies. In addition, tumour cells and tubulus cells of normal kidney demonstrated weak expression for CCR6. Occasionally, expression of CCR6 was observed by tumour-associated macrophages, CCR6-positive immature DCs were observed within T cell clusters of the tumour border zone (Figure 6e).

In contrast, lymphoid aggregates of the tumour border demonstrated a strong expression for CCR7 (Figure 6f). Within these clusters of CCR7-positive T cells numerous CD83+ mature DCs showed co-expression for CCR7 (Figure 7c). CCR6 expression by mature DCs could not be observed. As observed for CCR6 in some cases, weak expression for CCR7 could be observed by tumour cells and tubulus cells of normal kidney tissues as well. Tumour-associated macrophages showed no expression for CCR7.

## Discussion

A RCC is considered to be one of the most immunoresponsive cancers in humans. During the last few decades, promising new immunologic-based treatment strategies for this tumour entity have been developed [18]. However, the tumour response is observed only in a subset of patients. Thus, detailed knowledge of the mechanisms underlying the immune response at the interface between immune attack and immune suppression might help to improve the promising immunotherapeutic approaches in RCCs. More recently, emphasis has shifted to the use of DC vaccines for active immunization of cancer patients with *in vitro*-generated DCs, which have been fused with tumour cells or loaded with tumour antigens [19-21].

Different approaches of RCC vaccines have been explored in the metastatic and adjuvant setting in several studies [22,23]. Reviewing the current literature, about 20 non-randomised phase 1 or 2 immunotherapeutic trials have been published for DC-vaccines in metastasized RCCs [24-40]. As a summary, none of these patients developed significant treatment-related toxicity or autoimmunity-related side effects, while approximately 40% of the patients demonstrated clinical tumour regression [18]. Nevertheless, the results of DC vaccination in these non-randomised studies should be viewed with caution because of the relative small number of patients within each trial and the diversity of the vaccination strategies used. Although most groups currently use monocyte-derived DCs for clinical vaccine trials [41], the isolation procedures of the monocytes, the differentiation procedures towards DCs, and the loading and maturation procedures are heterogeneous between the published clinical studies [42]. Thus, the current data suggest that DC vaccination in patients with metastatic RCCs appears to be safe and results in a tumour-specific immune response which can achieve tumour regression in a significant subset of patients [18]. However, for efficient and reliable immunotherapy of tumours, the optimal protocol for DC-based vaccination remains to be clarified further. Therefore, detailed knowledge concerning the immune response in RCCs with special regard to the role of DCs *in vivo *is required for development of an optimal vaccination strategy. One very important issue in this context represents a detailed knowledge concerning the type and distribution of DC subsets, as well as the mechanisms underlying their migration, maturation, and function in RCCs. Recent data point to a significant role of DC subsets in Th1/Th2 polarization and the induction of the tumour immune response [14,43]. Thus, the determination of the origin and immune competence of tumour-associated DCs will be of great importance to our understanding of the development of tumour immunity for RCCs [44]. Therefore, we investigated the distribution and maturation of the different DC subtypes in RCCs and corresponding normal kidney tissues. The findings in the current study extend previous investigations for other tumour entities [14,15,45,46]. The results demonstrate a unique compartmentalization of immature and mature DCs within kidneys affected by RCCs.

All tumour samples displayed variable immature DC infiltration. Tumour-infiltrating immature DCs were heterogeneous, and two populations were identified. One population represented the CD1a+/Langerin+ Langerhans-cell type and the other type was CD1a+/Langerin- non-Langerhans cell. Since dermal (interstitial) DCs were found *in vitro *and *in vivo *to express CD1a, but not Langerin, we currently conclude that CD1a+/Langerin- tumour-infiltrating DCs represent immature interstitial DCs. The significance of this heterogeneity remains to be established. Earlier studies revealed that interstitial DCs generated *in vitro *from CD34+ precursors display more potent phagocytic activity than LCs [1,3].

The observed numbers of immature CD1a+ DCs in the tumour environment was much higher than in normal kidney tissues, suggesting increased homing and infiltration of immature DCs by the tumour. This finding was also been described by Troy et al. [47,48] nearly a decade ago. This may be best explained by the high levels of intratumoural MIP3-α, a chemokine, which is known to specifically attract immature DCs. Our results show an increased expression of MIP-3α by tumour cells of RCCs by RT-PCR and immunohistochemistry. The immunohistochemistry analysis with anti-MIP3-α is unfortunately not precise enough to allow a correlation between the amount of MIP3-α protein by the tumour cells and the amount of infiltrating immature DCs. However, our finding of an increased expression of MIP-3α mRNA in RCC cell lines, as well as in RCC tissue compared to mRNA derived from normal kidney, underlines a role for an increased expression of MIP-3α in homing or recruitment of immature DCs to RCC tissues. The increased number of immature DCs could also reflect a transient stage due to the high in-and-out migration or the sequestering of immature DCs within the tumour tissue. Another explanation might be tumour-induced maturation arrest of tumour-infiltrating DCs. Studies for different tumour entities have shown that in cancer patients, DCs in the blood, tumour tissues, and draining lymph nodes are often functionally defective and possess poor T cell stimulatory capacity [49]. One possible explanation for this observation might be the expression of tumour-derived factors, such as VEGF, TGF-β, and IL-10, which have been shown to inhibit differentiation or functional maturation of DCs [49-53]. Therefore, it has been suggested that immaturity of DCs in the tumour tissue may mediate tumour tolerance instead of immune activation caused by induction of T-cell anergy or favouring the development of regulatory T cells [54,55].

However, in contrast to the observed immaturity of intratumoural DCs, the more important finding might be the presence of numerous mature DCs accumulating specifically within the peri-tumoural areas. This finding suggests that additional factors might be involved in mature DC distribution. Because mature DCs are typically observed in lymphoid organs, where they closely interact with T cells, it is tempting to consider that their presence within the tumour tissue might reflect an ongoing tumour-specific immune response. Thus, the mature DCs could derive from any of the immature DC subset discussed above within the tumour tissues. The peri-tumoural localization of mature DCs observed in our study corresponds to observations in other tumour types, and RCC as well [15,47]. The preferential localization of mature DCs in lymphocyte-rich peri-tumoural areas of RCCs could be due to the resemblance of these areas to secondary lymphoid organs where mature DCs are normally found.

What instigates the mechanisms underlying the accumulation of mature DCs with a prominent T cell response in RCCs. Our results support the concept that specific homing of CCR7+ leucocytes to the tumour border may contribute to the anti-tumour response in RCCs, which at least partly resembles *de novo *development of a tertiary lymphoid tissue at the invasive margin of the tumours. Our results show a significant increased expression of lymphoid chemokines (SLC and MIP-3β) at the tumour border in contrast to tumour and normal kidney tissues. These results imply that the increased expression of SLC and MIP-3β may lead to a chemokine microenvironment normally observed in secondary lymphatic tissues. Expression of these chemokines favours homing and interaction of CCR7-expressing cells, such as mature myeloid DCs and naïve or memory T cells, facilitating their interaction for an optimal anti-tumour immune response. Thus, in analogy to secondary lymphoid organs, such as lymph nodes, chemokine-dependant co-localization of T-cells and antigen-presentation occurs at the tumour border, leading to the local generation of anti-tumour-specific T cells.

Interestingly, various studies have shown that the presence of a dense DC infiltration has been associated with prolonged survival and reduced incidence of metastases in patients with various human cancers, such as colorectal, gastric, esophageal, oral, and lung carcinoma [56-59]. In breast cancer, the number of CD83+ mature DCs, but not the number of CD1a+ or S100+ DCs, has been shown to be of prognostic relevance [60]. In light of their importance in anti-tumour immunity, surprisingly few studies have been aimed at the presence of DCs and its potential clinical correlates in RCCs. For interferon pre-treated RCCs a trend toward a better outcome and better prognosis has been found in patients with a higher number of S100+ DCs within the tumour tissue [61,62]. Although S100 protein has long been used to indicate DCs, its significance is still under confusion. In our experiments, we found S100 protein to be co-expressed by numerous CD68+ macrophages. Thus, S100 immunohistochemistry was not suitable for evaluation of DC numbers in RCCs (data not shown). In a more recent study, Kobayashi et al. [63] investigated whether or not the local immune environment might be associated with the tumour response following treatment with interferon-α and interleukin-2 in RCCs. Their results demonstrated a higher number of mature CD83+ DCs in tumour tissue of responders following cytokine treatment. In addition, the responders survived longer than non-responders. In contrast, other tumour-associated immune cells, such as CD8+ T-cells or tumour-associated macrophages, were not associated with treatment response or survival outcome. Thus, the presence of high number of CD83+ mature DCs might represent a predictive factor for the clinical outcome in patients with RCCs [63].

## Conclusions

These and our observations suggest that the presence of an "immunologic functional unit" in RCCs consisting of antigen-presenting DCs and activated T cells at the tumour border might represent prognostic significance. In this scenario the combination of a low number or absence of DCs with a low density of activated T cells would predict a worse outcome, while a high density of mature DCs and activated T cells predicts a favourable disease outcome. This would also suggest that the presence of mature antigen-presenting DCs at the primary site together with that of activated T cells, represents a functional immune response against RCC progression. Thus, our observations open new insight regarding DC involvement and function concerning the anti-tumour response in RCCs. However, the limited number of RCC tumour samples analyzed in this study and the short follow-up times does not allow us to establish a prognostic significance of the infiltration of tumour by immature or mature DCs. Such determination will require the analysis of a large number of samples in prospective or retrospective studies. Therefore, further analysis of the APC system will provide clues to the understanding of tumour immunity as well as improvement of the efficiency of vaccine strategies in RCCs and other immunogenic tumour entities.

## Abbreviations

RCC: renal cell carcinoma; DC: dendritic cell; APC: antigen-presenting cell

## Competing interests

The authors declare that they have no competing interests.

## Authors' contributions

PM performed the design of the study and the statistical analysis. PM and SB performed the experimental investigations. WM participated in the writing of the manuscript. HJR participated in conception of the idea and writing of the manuscript. All authors read and approved the final manuscript.

## Pre-publication history

The pre-publication history for this paper can be accessed here:

http://www.biomedcentral.com/1471-2407/10/578/prepub
